# Genetic variants in CYP2B6 and CYP2A6 explain interindividual variation in efavirenz plasma concentrations of HIV-infected children with diverse ethnic origin

**DOI:** 10.1371/journal.pone.0181316

**Published:** 2017-09-08

**Authors:** Sandra Soeria-Atmadja, Emma Österberg, Lars L. Gustafsson, Marja-Liisa Dahl, Jaran Eriksen, Johanna Rubin, Lars Navér

**Affiliations:** 1 Department of Pediatrics, Karolinska University Hospital, Stockholm, Sweden; 2 Department of Clinical Science, Intervention and Technology (CLINTEC), Karolinska Institutet, Stockholm, Sweden; 3 Department of Laboratory Medicine, Division of Clinical Pharmacology, Karolinska Institutet, Karolinska University Hospital, Stockholm, Sweden; Stellenbosch University, SOUTH AFRICA

## Abstract

**Background:**

Approximately 2.6 million children live with HIV globally, and efavirenz (EFV) is one of the most widely used antiretroviral agents for HIV treatment in children and adults. There are concerns about the appropriateness of current EFV dosing and it has been discussed whether EFV dosing should be adapted according to genotype in children as suggested for adults.

**Aim:**

To investigate if pediatric EFV dosing should be guided by genetic variation in drug metabolizing enzymes rather than by body weight.

**Method:**

EFV plasma concentrations measured for clinical purposes from all children less than 18 years old at Karolinska University Hospital, Stockholm, Sweden, treated with EFV were collected retrospectively. They were genotyped for eleven polymorphisms in genes coding for drug-metabolizing enzymes and P-glycoprotein, of potential importance for EFV disposition. Data on country of origin, sex, age, weight, HIV RNA, viral resistance patterns, CD4 cells, adherence to treatment, subjective health status and adverse events were collected from their medical records.

**Results:**

Thirty-six patients and 182 (mean 5 samples/patient) EFV plasma concentration measurements from children of African, Asian and Latin American origin were included. EFV plasma concentration varied 21-fold between measurements (n = 182) (0.85–19.3 mg/L) and 9-fold measured as mean EFV plasma concentration across the subjects (1.55–13.4 mg/L). A multivariate mixed-effects restricted maximum likelihood regression model, including multiple gene polymorphisms, identified CYP2B6*6 T/T (p < 0.0005), CYP2B6*11 G/G (p < 0.0005), CYP2A6*9 A/C (p = 0.001) genotypes, age at treatment initiation (p = 0.002) and time from treatment initiation (p < 0.0005) as independent factors significantly related to log_e_ concentration/(dose/weight). The contribution of the model to the intra- and interindividual variation were 6 and 75%, respectively (Bryk/Raudenbush R-squared level).

**Conclusion:**

Genetic polymorphisms in CYP2B6 and CYP2A6 explained a significant proportion of variability in EFV plasma concentration in HIV-infected children in a multi-ethnic outpatient clinic. Knowledge about individual variants in key drug metabolizing enzyme genes could improve clinical safety and genotype directed dosing could achieve more predictable EFV plasma concentrations in HIV-infected children.

## Background

World-wide approximately 2.6 million children live with human immunodeficiency virus type 1 (HIV-1) [1]. The number is slowly decreasing due to increasing access to mother-to-child prevention programs. The reverse transcriptase inhibitor efavirenz (EFV) in combination with two other antiretroviral agents is first line treatment, recommended by WHO and the most widely used HIV-drug in children and adults globally. There are concerns about the appropriate EFV dosing schedules and treatment results in both adults and children [2–9]. Pediatric studies have found both under- [2–4, 9] as well as overdosing [8, 9] to be of concern, whereas in adults, most attention is paid to the risk for overdosing [6, 7]. In children, dosing schedules are complex as changes in drug metabolic capacity related to age and increasing body weight need to be taken in account.

EFV is primarily metabolized in the liver by cytochrome P450 2B6 (CYP2B6) [10] and to a lesser extent by CYP3A4, CYP3A5, CYP2A6 and CYP1A2 and other drug-metabolizing enzymes such as UDP-glucuronosyltransferase 2B7 (UGT2B7) [8, 9, 11–16]. The CYP2B6 gene is highly polymorphic, and several functional polymorphisms causing decreased enzyme activity have been reported to influence EFV plasma concentrations in adult patients of different ethnic origins [9, 11–17]. A similar impact of polymorphisms on EFV plasma concentration has been indicated in studies in children [9, 18, 19]. A polymorphism causing expression of the hepatic CYP3A5 enzyme is more frequent in African than in Caucasian populations [20, 21] and variability in the expression of the gene ABCB1, which encodes P-glycoprotein, an ATP-dependent drug efflux pump responsible for drug transport across extra- and intra-cellular membranes may also contribute to variable plasma EFV concentrations [13, 22]. The plasma concentrations of EFV show interindividual variation in adults [23] to which genetic polymorphisms in drug metabolizing enzyme genes contribute. Studies in pediatric populations [8, 9, 18, 19, 24–27] are increasing, but few have assessed the relative importance of different polymorphisms for the overall variability in EFV concentrations in children. Genetic polymorphisms show interethnic differences in frequency across the globe and few adult studies are conducted in multi ethnic settings [28]. Such pediatric studies are even more rare.

In adults, the risk of central nervous system (CNS) toxicity of EFV is concentration dependent [29, 30], most evident during the first weeks of therapy. Later, side effects are more subtle, probably because of tolerance development. One recent case report relates CNS adverse effects to aberrant EFV metabolism and CYP2B6 genetic variation in children [24]. The risk of therapeutic failure in adults is reported to increase with levels < 1.0 mg/L in drug naïve adult patients [31] and at levels < 2.2 mg/L in adults with expected suboptimal efficacy of NRTIs [23]. Children who initiated a first-line EFV-based regimen and had a minimum EFV concentration > 1.1 mg/L had to a higher extent a 2 log_e_ decrease in HIV RNA copies/mL, compared to those with lower EFV plasma concentration [32].

The standard adult EFV dose is 600 mg once daily, but recently a clinical study [6] showed an equal effect and a slightly higher tolerability using 400 mg, that since 2015 is an alternative recommended option for first-line treatment of HIV in adults by WHO [33]. Genotype specific dosage regimens have been suggested to be relevant for pediatric African populations [27, 34] but pharmacokinetic data of EFV in children are scanty [9, 18, 19, 27].

The half-life of EFV is long, approximately 40–55 hours [5, 35], which makes dosage once daily possible. For the same reason, the timing of monitoring the mid-interval EFV concentration does not necessarily have to be exact [36].

We report result from the pediatric HIV outpatient clinic at Karolinska University Hospital, where plasma EFV concentrations have been analyzed for clinical purpose since June 2005. The plasma concentrations were related to polymorphisms in genes coding for drug metabolizing enzymes, for the transporter P-glycoprotein and other factors with known or suspected effect on plasma levels of EFV. The aim was to explore if EFV treatment could be optimized in children using genetic information in a multi-ethnic patient group.

## Materials and methods

### Ethics

Written informed consent from the patients and/or their legal guardians was obtained and documented in the participants’ medical records. All participants were provided with written and oral information about the study. The study was approved by the Regional Ethical Review Board in Stockholm, Sweden (No: 2012/1696-31/1).

### Subjects

All patients at the pediatric outpatient clinic at Karolinska University Hospital, Stockholm, Sweden with ongoing or previous treatment with EFV before the age of 18 between June 2005 and October 2013 were approached. All 43 patients matching the inclusion criteria accepted participation. The patients at the clinic represent a diversity of ethnic origin, probably with a high degree of genetic variations.

### Methods

#### Dosing

The dosing of EFV in children ≥ 3 years of age followed the Pediatric European Network for Treatment of AIDS (PENTA) guidelines [37] and are shown in Table 1. We combined 50 mg, 200 mg and 600 mg tablets to achieve an adequate start dose.

**Table 1:** Summary of administration information for efavirenz (Stocrin).

13–15 kg	200 mg o.d.
15–20 kg	250 mg o.d.
20–25 kg	300 mg o.d.
25–32.5 kg	350 mg o.d.
32.5–40 kg	400 mg o.d.
Over 12 years and/or ≥ 40 kg	600 mg o.d.

Tablets: 50 mg, 200 mg and 600 mg.

Bedtime dosing and intake without food were recommended.

o.d.; once daily

#### Sampling

Plasma concentrations of EFV were analyzed between June 2005 and October 2013 as part of the clinical follow-up at the outpatient clinic for HIV-infected children at the hospital. The first sampling was recommended to be performed 2 to 3 weeks after treatment initiation. The recommendation was to take EFV in the evening. EFV plasma concentration, sampled 14 to 20 hours after intake was analyzed. We collected blood samples for genotyping together with routine sampling at an ordinary follow-up visit. Plasma EFV concentrations and genotypes were analyzed at the Department of Clinical Pharmacology, Karolinska University Hospital.

Dose adjustments were made based on plasma EFV concentration levels to reach the suggested target concentration range of 1.0 to 4.0 mg/L, 14–20 hours after drug intake [36]. Dose adjustments were also made if adverse effects were suspected.

#### Efavirenz analysis

Venous blood samples were collected in 4 mL Na-heparin tubes and centrifuged at 1000g within 2 hours. An aliquot of 500 μL plasma was transferred to plastic tubes and stored at -20°C until analysis.

The bioanalytical method for quantification of EFV was based on protein precipitation followed by liquid chromatography and UV-detection (HPLC-UV) as described [14]. In brief a 100 μL aliquot of each sample was transferred to a glass vial and precipitated with 200 μL acetonitrile. They were vortexed for 10 seconds and then centrifuged for 3 min at about 800g. A 6 μL aliquot of the supernatant was injected onto an Agilent 1100 HPLC-UV system (Agilent, Santa Clara, CA) equipped with a Luna reversed phase column (2.5 μm. 50 × 2 mm. Phenomenex, Torrance, CA). Separation was achieved by isocratic elution with a mobile phase consisting of v/v 30% methanol, 30% acetonitrile and 40% MQ water and 10 mmol/L acetic acid and 4 mmol/L KOH at a flow rate of 0.8 ml/min. EFV was quantified at λ 210 nm at 11 min after injection. Method calibrators and controls were prepared in-house by spiking drug free human plasma with appropriate amounts of EFV (EDQN, Strasbourg, France; spiking solutions prepared in MQ water), spiking volume not exceeding 2% final volume. The quantification range was 0.158 to 31.6 mg/L. The method performance was monitored by analysis of internal quality control samples and participation in the KKGT (Kwaliteitsbewaking Klinische Geneesmiddelanalyse en Toxicologie, Den Haag, NE) antiretroviral proficiency testing scheme. Precision expressed as coefficient of variation (CV) at 2.5 mg/L was 5.7%.

We evaluated all available plasma EFV samples. A sample was excluded if poor adherence to treatment was verified or suspected on a particular date or if the interval after the last administered dose was outside the interval 14 to 20 hours. Likewise, if EFV was administered as a syrup the corresponding EFV plasma concentration values were excluded, due to different pharmacokinetic characteristics.

The suggested recommended therapeutic interval for adult patients on EFV is 1.0 to 4.0 mg/L [36]. This interval also guides clinical care in the pediatric outpatient clinic.

EFV plasma concentration divided by (dose/weight) was used as the main outcome measure in order to adjust for differences that could influence the EFV plasma concentration during the course of treatment and between individuals, such as weight and dosage changes based on EFV plasma levels.

#### Genotyping

Genomic DNA was isolated from 1.5 mL whole blood collected in EDTA using QIAamp DNA MiniKit (QIAGEN GmbH, Hilden, Germany) and stored at -20°C until analysis. Genotyping was carried out with validated TaqMan assays from Life Biotechnologies (Thermo Fisher Scientific) according to the manufacturer’s guidelines and a StepOnePlus Real-Time PCR system (Life Technologies). The literature was reviewed for polymorphisms in drug metabolizing enzyme genes and ABCB1 to identify polymorphisms of potential importance for EFV pharmacokinetics in different ethnic populations. The following eleven polymorphisms in CYP2A6, CYP2B6, CYP3A4, CYP3A5 and ABCB1 genes were selected and analyzed: CYP2A6*9 A>C (rs28399433, C_30634332_10), CYP2B6 g.18492 C>T (rs2279345, C_26823975_10), CYP2B6*6 G>T (rs3745274, C_7817765_60), CYP2B6*11 A>G (rs35303484, C_33845811_20), CYP2B6*18 C>T (rs28399499, C_60732328_20), CYP3A4*22 G>A (rs35599367, C_59013445_10), CYP3A5*3 T>C (rs776746, C_26201809_30), CYP3A5*6 C>T (rs10264272, C__30203950_10), CYP3A5*7 insertion A (rs41303343, C__32287188_10), ABCB1 c.3435 A>G (rs1045642, C_7586657_20), andABCB1 c.4036 C>T (rs3842, C_11711730_20).

#### T-cell populations and viral load

Analyses of CD4^+^ and CD8^+^ T-cell counts and plasma HIV-1 RNA load were part of the clinical routine using flow cytometry and Cobas Amplicor (Roche Molecular Systems Inc., Branchburg, New Jersey, USA), respectively. The lowest detection limit was 50 copies/mL before June 2007, and thereafter 20 copies/mL.

#### Clinical and laboratory data

Clinical information and laboratory results (sex, age, ethnicity, weight, reported adverse effects, EFV dose, EFV plasma concentration, CD4-cell count, HIV RNA load and routine laboratory parameters) were obtained from the medical records (Take Care) and the Swedish HIV quality register (InfCare HIV) [38]. Data were extracted for each patient when the variables weight, dose, EFV plasma concentration, HIV RNA and CD4-cell count were simultaneously recorded. Subjective health status and reported adverse effects, if any, were also obtained from the medical record on the same occasion. Adherence to the treatment regimen was assessed through HIV-RNA viral load, viral resistance patterns and CD4-cell counts, and from the patients’ history of adherence stated in the medical records. When time between weight registration was longer than 16 weeks (± 8 weeks) a mean weight was calculated based on the two closest available weights. Once a patient’s body weight was considered equal to adult weight, one single weight measurement was accepted.

#### Data analysis

We used descriptive statistics, linear regression, Student t-test, Fishers exact test, linear regression analysis and a mixed-effects REstricted Maximum Likelihood (REML) regression model [39] and Bryk/Raudenbush R-squared level [40] to describe and correlate weight, dose, sex, age and gene polymorphisms to EFV plasma concentrations. Separate mixed models for all genetic polymorphisms adjusted for age, sex and time from treatment initiation were ran. The significant variables were then analyzed using a multivariate mixed model. The outcome measure EFV plasma concentration/(dose/weight) was log_e_ transformed to normalize the distribution. We could not perform statistical analyses of ethnic origin due to small numbers in certain groups.

We used the software JMP 12.1.0. SAS Institute Inc., Cary. USA and Stata version 13.1, Statacorp, Texas, USA. P-values < 0.05 were considered to be significant and were calculated two-sided. Statistical power was not calculated, as the study was retrospective and the population studied consisted of all available patients at the clinic.

## Results

### Subjects

Out of the 43 patients that matched the inclusion criteria and accepted participation a total of seven patients were excluded; two did not have available EFV plasma concentrations, four took their medication in the morning instead of following the standard practice of administration of EFV in the evening and one patient took EFV only as a syrup which affects comparison. The remaining 36 patients were included in the study. The total number of EFV plasma concentration values registered was 237, of which 182 (1–11 samples/patient, mean 5.1) were included in the analyses. Fifteen EFV plasma concentrations were excluded since EFV was administered as a syrup, 15 due to suspected or verified poor compliance and 25 were excluded from patients taking EFV in the morning.

Thirty out of 36 (83%) patients were of African origin, four (11%) of Asian origin, and two (6%) were from Latin America. The countries of origin were Burundi, Eritrea, Ethiopia, Sudan, Ivory coast, Kenya, Liberia, Rwanda, Somalia, Tanzania and Uganda constitutes African origin; India, Philippines, Thailand and Uzbekistan constitutes Asian origin; Chile and Honduras constitutes Latin American origin.

The genotype distribution and minor allele frequencies in the cohort of the 36 included patients are given in Table 2.

**Table 2:** Genotype and allele frequency distribution.

	Genotype	Origin			Total	Frequency %
		African	Asian	Latin American		
CYP2A6*9 (rs28399433)	**AA**	26	2	1	29	81
** **	**AC**	4	1	1	6	17
** **	**CC**	0	1	0	1	3
**Minor allele**	**C**	4	3	1	8	11
**CYP2B6 g.18492 (rs2279345)**	**CC**	24	1	2	27	75
** **	**CT**	6	3	0	9	25
** **	**TT**	0	0	0	0	0
**Minor allele**	**T**	6	3	0	9	13
CYP2B6*6 (rs3745274)	**GG**	10	4	1	15	42
** **	**GT**	15	0	1	16	44
** **	**TT**	5	0	0	5	14
**Minor allele**	**T**	25	0	1	26	36
CYP2B6*11 (rs35303484)	**AA**	28	4	2	34	94
** **	**AG**	1	0	0	1	3
** **	**GG**	1	0	0	1	3
**Minor allele**	**G**	3	0	0	3	4
CYP2B6*18 (rs28399499)	**CC**	0	0	0	0	0
** **	**CT**	4	0	1	5	14
** **	**TT**	26	4	1	31	86
**Minor allele**	**C**	4	0	1	5	7
CYP3A4*22 (rs35599367)	**AA**	0	0	0	0	0
** **	**AG**	2	0	0	2	6
** **	**GG**	28	4	2	34	94
**Minor allele**	**A**	2	0	0	2	3
CYP3A5*3 (rs776746)	**CC**	11	2	1	14	39
** **	**CT**	13	2	1	16	44
** **	**TT**	6	0	0	6	17
**Minor allele**	**T**	25	2	1	28	39
CYP3A5*6	**CC**	22	4	2	28	78
	**CT**	7	0	0	7	19
	**TT**	1	0	0	1	3
**Minor allele**	**T**	9	0	0	9	13
CYP3A5*7	**A/A**	0	0	0	0	0
	**A/-**	2	0	0	2	6
	**-/-**	28	4	2	34	94
**Minor allele**	**A**	2	0	0	2	3
**ABCB1 (rs1045642)**	**AA**	1	1	1	3	8
** **	**AG**	8	3	0	11	31
** **	**GG**	21	0	1	22	61
**Minor allele**	**A**	10	5	2	17	26
**ABCB1 rs3842**	**CC**	3	0	0	3	8
** **	**CT**	8	1	1	10	28
** **	**TT**	19	3	1	23	64
**Minor allele**	**C**	14	1	1	16	22

The distribution of alleles between the 11 studied genotypes.

### Efavirenz treatment

The median age at treatment initiation was 9 years (range 2–17 years) and the median duration of EFV-based therapy during the study was 48 months (1–121). In 12 patients (33%), the dosage of EFV was adjusted during the course of treatment based on measured EFV plasma concentrations. For different reasons 13 patients (36%) ended their EFV therapy during the study period. Reasons for ended EFV therapy were suspected adverse effects (n = 5), viral resistance to EFV (n = 6) and other reasons for change to other antiretroviral regimen (n = 2). In total, results from 49 viral resistance analyses from 23 patients were available.

### Association between dose/weight and efavirenz plasma concentration

The first available EFV plasma concentrations (n = 36), taken 12 to 1996 (median = 35) days after treatment initiation were investigated for associations between the recommended dose/weight and the suggested recommended therapeutic interval of EFV plasma concentration, 1.0–4.0 mg/L. These concentrations represented the initial dosage for the patients included and were taken before any dose adjustments and in some cases before enzyme induction. In 34 (94%) of the patients the dose/weight corresponded to recommendations and 24 of those (71%) did achieve an EFV plasma concentration within the recommended therapeutic interval.

In a linear regression model the initial dose/weight did not correlate to the first measured EFV plasma concentration in each patient (p = 0.63).

**Fig 1:**
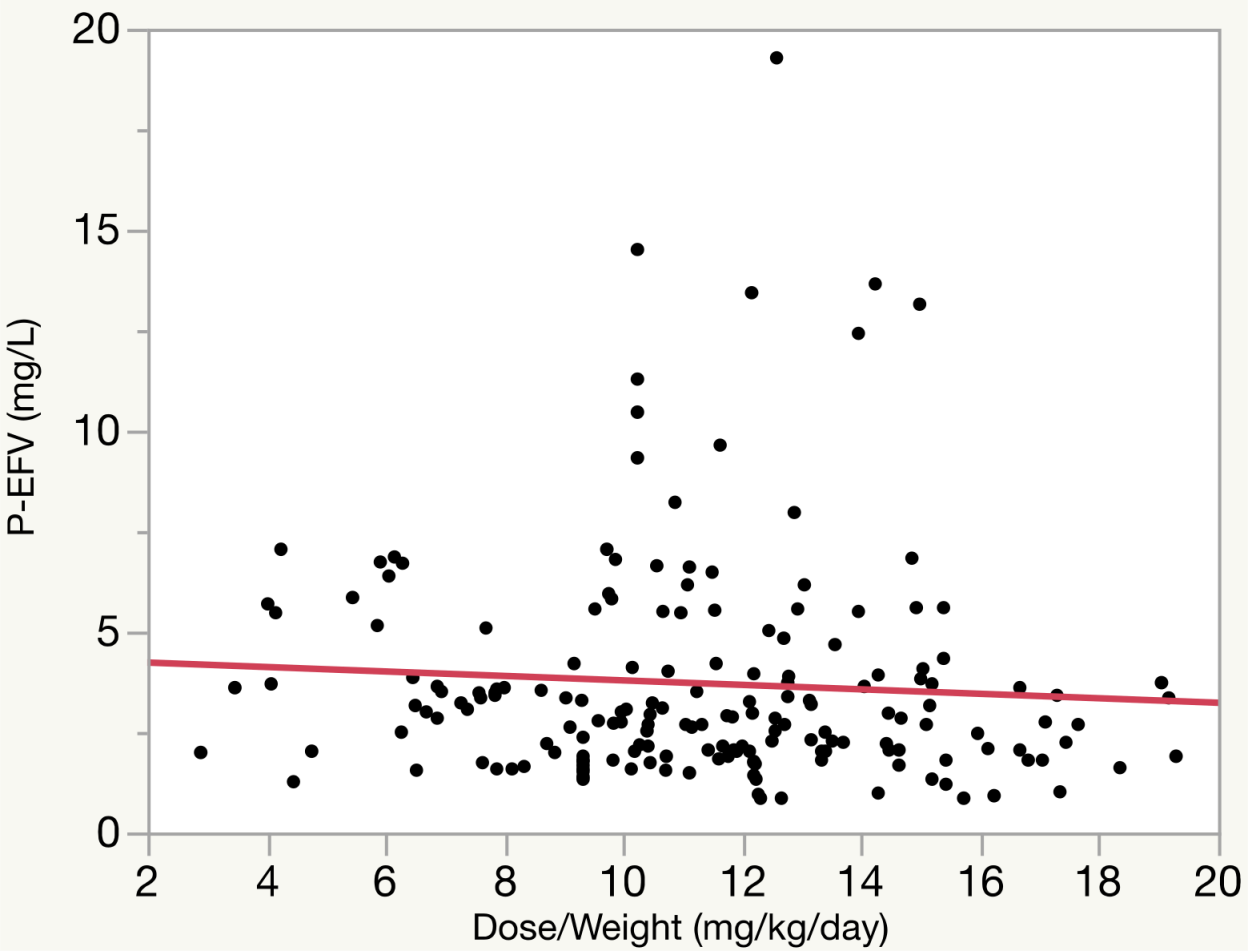
Correlation between dose/weight and efavirenz plasma concentration. Scatterplot visualizing the EFV plasma concentrations from 36 patients with 182 samples and corresponding dose/weight. Linear regression with cluster robust standard errors: P-EFV (mg/L) = 4.34–0.0556*Dose/Weight (mg/kg/day); p = 0.36; r^2^ = 0.0046.

A scatterplot and linear regression analysis of all dose/weight values (36 patients, 182 samples) and the corresponding EFV plasma concentrations shows no relationship (p = 0.36, r^2^ = 0.0046) (Fig 1).

### Factors influencing log_e_ efavirenz plasma concentration/(dose/weight)

The association between genetic variants of CYP2A6, CYP2B6, CYP3A4, CYP3A5, and ABCB1 and the log_e_ EFV concentration/dose/weight were analyzed in a mixed-effects REML regression model. When analyzing one gene at a time, adjusted for age, sex and time from treatment initiation, we found significant association between log_e_ EFV concentration/(dose/weight) and CYP2A6*9 C/C (p = 0.017), CYP2B6 g.18492 C/T (p = 0.009), CYP2B6*6 T/T (p < 0.0005) and CYP2B6*11 G/G (p = 0.032) genotypes. No association was found for the polymorphisms in CYP3A4, CYP3A5 or ABCB1.

A mixed-effects REML regression model that included multiple polymorphisms, identified CYP2B6*6 T/T (p < 0.0005), CYP2B6*11 G/G (p < 0.0005), CYP2A6*9 A/C (p = 0.001) genotypes as independent factors with a significant positive correlation to log_e_ EFV plasma concentration/(dose/weight) as shown in Table 3.

**Table 3:** Relation between genetic variants, age at treatment initiation, time from treatment initiation, sex and log_e_ EFV plasma concentration/(dose/weight).

	Coefficient	P > |z|	[95% Confidence Interval]	
CYP2B6*6 G/T genotype	0.053	0.74	-0.26	0.36
CYP2B6*6 T/T genotype	1.10	< 0.0005	0.67	1.54
CYP2B6*11 A/G genotype	0.54	0.17	-0.24	1.32
CYP2B6*11 G/G genotype	1.60	< 0.0005	0.86	2.33
**CYP2B6** g.18492 **C/T genotype**	-0.084	0.61	-0.41	0.24
CYP2A6*9 A/C genotype	0.50	0.001	0.20	0.80
CYP2A6*9 C/C genotype	-0.66	0.12	-1.49	0.16
**Time from treatment initiation**	0.00014	< 0.0005	0.000074	0.00021
**Age at treatment initiation**	0.064	0.002	0.023	0.10
**Sex**	0.084	0.54	-0.19	0.36

First, separate mixed models for all genetic polymorphisms adjusted for time from treatment initiation, age and sex were run. The significant variables were then analyzed in a multivariate mixed model. The outcome measure EFV plasma concentration/(dose/weight) was log_e_ transformed to normalize the distribution.

The REML regression model explained according to Bryk/Raudenbush R-squared Level 1, six percent of the intraindividual variation and according to Bryk/Raudenbush R-squared Level 2, 75% of the interindividual variation. The corresponding Bryk/Raudenbush R-squared Level 1 and 2 for each variable in the univariate mixed model were 0.2% and 21% for CYP2B6*6, -0.05% and 11% for CYP2B6*11 and -0.5% and 14% for CYP2A6*9, respectively.

Both age at treatment initiation (p = 0.002) and time from treatment initiation (p < 0.0005) showed a significant positive correlation to log_e_ EFV plasma concentration/(dose/weight) in the REML regression model. There was no significant relation between sex (p = 0.54) and log_e_ EFV concentration/(dose/weight) (Table 3).

**Fig 2:**
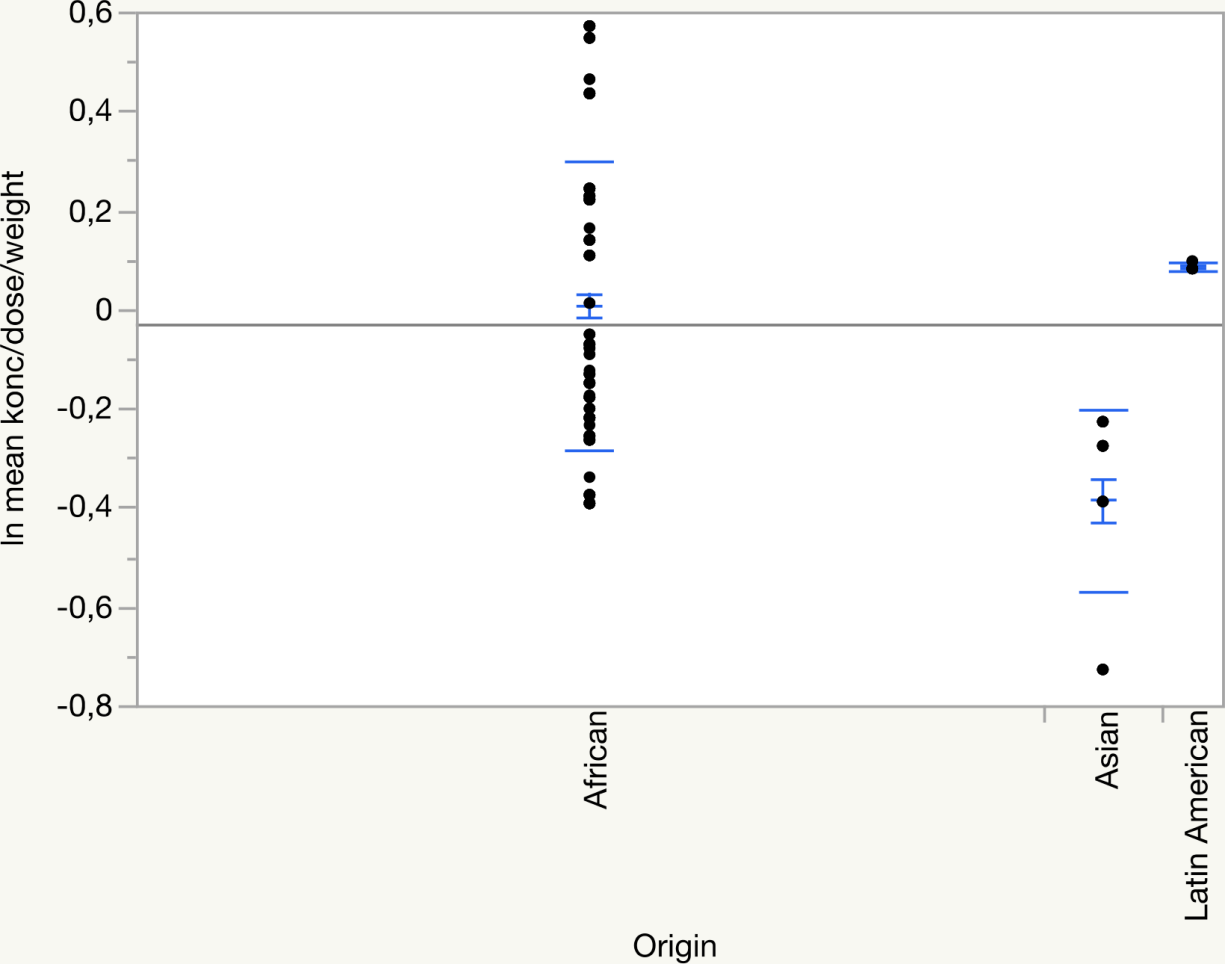
Distribution of log_e_ mean efavirenz plasma concentration/(dose/weight) by geographical origin. Number of patients 36 (African 30, Asian 4, Latin American 2), number of samples 182. Mean, standard deviation and 95% confidence interval are incorporated.

The distribution of log_e_ mean efavirenz plasma concentration/(dose/weight) by origin is illustrated in Fig 2.

### Variations in EFV plasma concentration

**Fig 3:**
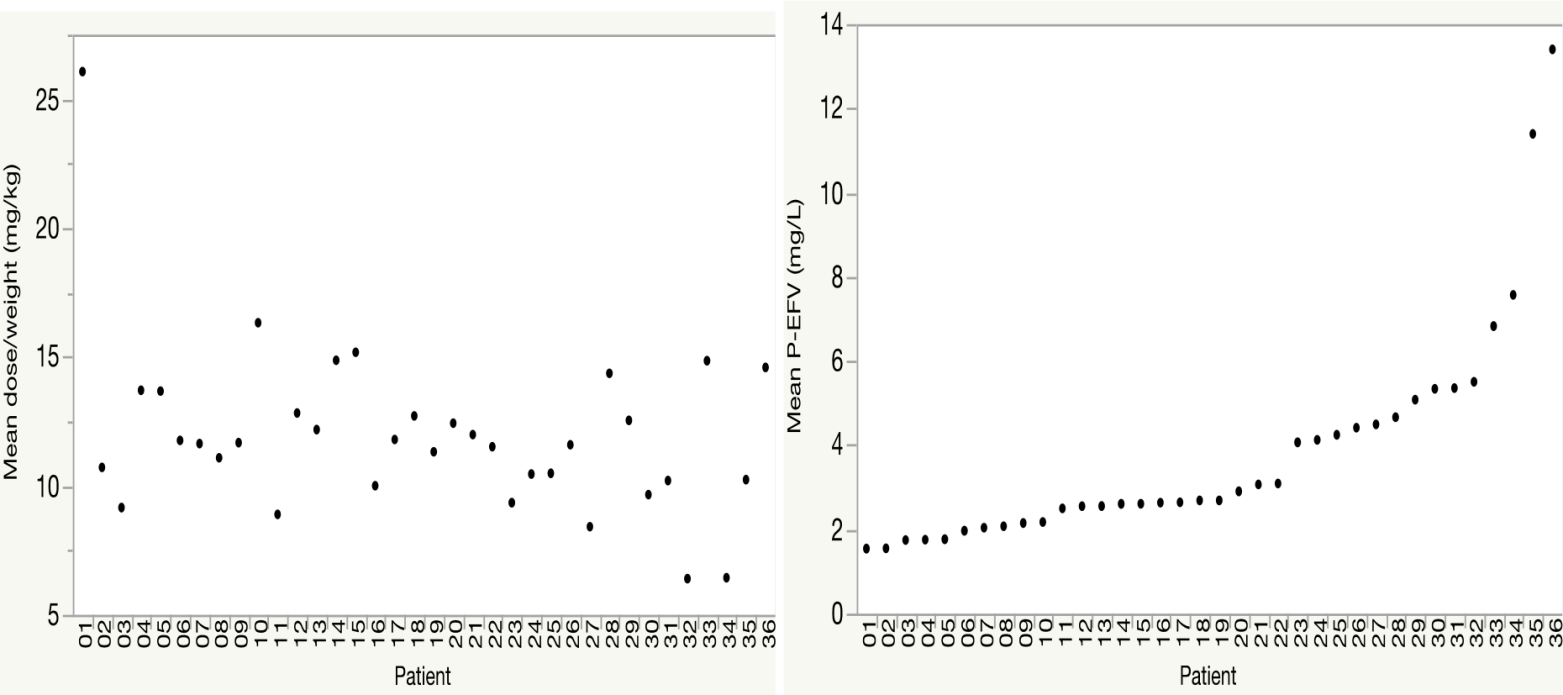
Interindividual variability in mean EFV plasma concentrations and mean dose/weight. Variation in the mean dose/weight (left) and EFV mean plasma concentration (right) in all included patients (n = 36).

Considerable inter- and intraindividual variations in EFV concentrations were observed. The dose and weight variables differ between individuals and during the course of treatment, and may contribute to the interindividual variation in EFV plasma concentration. Fig 3 clearly shows that EFV plasma concentration differ greatly between patients even when the dose/weight is similar.

Four patients (seven samples) had occasional sub therapeutic concentrations at least once during the study. Fifteen patients (48 samples) had supratherapeutic concentrations and 32 patients (127 samples) had at least one EFV plasma concentration within the recommended therapeutic interval.

### Reported adverse events

Eleven (31%) of the 36 studied patients ended their EFV therapy and five did so because of suspected adverse effects. Adverse events were reported at some point during the studied period with EFV treatment by 19 patients (53%). No persistent abnormal routine laboratory tests were observed among the included patients. The total number of reported adverse events was 31. These were grouped into eleven different types: Nausea, malaise, stomach pain (transient) (n = 7), other gastrointestinal complaints (transient) (n = 2), lightheadedness, vertigo, unsteadiness (transient) (n = 3), weariness, fatigue, tiredness (transient) (n = 2), affected cognition, loss of concentration, focus, etc. (n = 3), headache (transient) (n = 3), disrupted sleep patterns, nightmares, insomnia (transient) (n = 3), skin rash—(nettle-rash/eczema) (transient) (n = 3), gynecomastia (n = 4) and hyperlipidemia (n = 1). Most events reported were related to transient CNS symptoms (n = 14), followed by transient gastrointestinal events (n = 9). Twenty-three of the 31 reported adverse events (74%) were transient and occurred in the first weeks of EFV treatment. No severe adverse events related to EFV, such as admission to hospital, intensive care or death, were observed.

### Clinical effect of EFV treatment

The last registered viral load during EFV therapy was < 50 copies/mL in 28 patients and > 50 copies/mL in eight patients. There was no difference in treatment efficacy measured as viral load above or below 50 copies/mL related to mean EFV plasma concentration (Fisher exact test, p = 1.0) or to the occurrence of side effects (p = 0.26).

## Discussion

Interindividual variability in EFV plasma concentrations is well known in adults and was confirmed in this pediatric study in accordance with recent findings in children [8, 9, 18, 19, 24–26]. As previously described there are concerns regarding the low degree of association between weight, age and EFV plasma concentration, which serve as basis for today’s treatment recommendations [37]. We found no correlation between dose/weight and EFV plasma concentration in this study of HIV-infected children almost exclusively being immigrants from high prevalence areas. Most previous pharmacogenetic studies have been performed in more homogenous settings [8, 9, 26]. Our findings support that genotyping could serve as a tool to achieve better dosing and prediction of EFV plasma concentration in a pediatric multiethnic setting.

There is a need for useful pediatric information about optimal EFV dosing related to pharmacogenetics, ethnicity and age. Pediatric studies have found both over- [8, 9] as well as underdosing [2–4, 9] to be of concern. These studies included different parameters and the results are partly conflicting which make them difficult to compare and draw conclusions from. One study found the liquid formulation and the current dosing guidelines to result in subtherapeutic concentrations in children carrying the CYP2B6-516-G/G genotype [3]. In another study, not analyzing genotype, African children aged 3–12 years, efavirenz dosed according to 2006 WHO/manufacturer's recommendations, had lower and highly variable efavirenz PK parameters compared with adult data from manufacturer's leaflet [2]. A third study found a significant number of both over- and underdosing in a cohort of South African children and constructed a dosing model including age, weight and CYP2B6 G516 genotype to optimize EFV plasma concentrations [9]. Evaluation of pediatric studies must consider differences in drug formulations (pills vs. syrup) [3] and dosing recommendation as well as ethnicity and age. The use of EFV has been extended to ages 3 months to 3 years in the United States. Recently CYP2B6 genotype-directed dosing was considered to be required for optimal efavirenz exposure in this age group [41]. Studies in adults have reported differences related to ethnicity with higher EFV plasma concentrations in Hispanics and Africans than in Caucasians [42–44], probably related to interethnic differences in occurrence of different polymorphisms in drug metabolizing enzymes. Pediatric information about the importance of ethnicity on EFV plasma concentration is scarce. The children of Hispanic or Asian origin in our study were few, why performing analyses between ethnical groups was difficult. However, an adult study [7] found both gender and race, but not CYP2B6 C1459T polymorphism to be important factors for variability in plasma efavirenz concentrations.

A mixed-effects REML regression model, including multiple genes, identified CYP2B6*6 T/T, CYP2B6*11 G/G, CYP2A6*9 A/C genotypes, age at treatment initiation and time from treatment initiation, as independent factors significantly correlated to log_e_ EFV plasma concentration/(dose/weight) (Table 3). The Mixed model REML regression model explained as much as 75% of the interindividual variation in EFV plasma concentration. The contribution of the studied polymorphisms to the interindividual variance (Bryk/Raudenbush R-squared 2) followed the pattern CYP2B6*6 (21%) > CYP2A6*9 (14%) > CYP2B6*11 (11%) which is in line with a previous report that failed to find a substantial role for *11, but instead for *18 [19]. The model should be interpreted with caution as the degree of explanation tends to increase with the number of variables added to the model [40], but has the advantage of providing an estimation of the contribution of genetic variants for interindividual variance in EFV plasma concentrations. To the best of our knowledge such data is lacking in the literature for children.

The intraindividual variation could not be explained by the model. Variation in sampling time after dosage, intake with or without food, poor adherence and enzyme induction are factors that are known to affect intraindividual and interindividual variation in EFV plasma concentration. All these factors could affect the results.

We did not find any difference in EFV plasma concentrations related to sex, which is consistent with previous reports [9, 35]. The apparent peripheral volume of distribution has been shown to be twofold higher in women compared to men [14]. This difference is not expected in children but is a possible explanation of differences in EFV plasma concentrations between adult males and females [7].

In the REML regression model both older age at treatment initiation (p = 0.002) and time after treatment initiation (p < 0.0005) were significantly positively correlated to log_e_ EFV concentration/(dose/weight). This suggests that the EFV plasma concentration increases with age, regardless of changes in weight and dosage and would also mean that lower dose/weight yields the same EFV plasma concentration at an older age, which is in line with the current dosage recommendation. This result conflicts the expected increased plasma clearance due to enzyme induction that is known to occur in adults but is not well-defined in children [13]

Fifty-three percent of the patients reported adverse events and 71% of the events reported were transient, occurring in the first weeks of EFV treatment as described [45]. The majority were CNS-related. When studying adverse effects in retrospect, there is a risk of missing adverse drug effects not reported by patients and adverse drug effects such as suspected cognitive symptoms or poor ability to concentrate, are difficult to interpret since account has to be taken to the normal variation of the population. The fact that many of the studied patients are refugees, have lost parents and are experiencing complex socioeconomic situations is a complicating factor as well when considering the possible contribution of EFV to neurocognitive problems. However, some correlation between adverse effects and EFV plasma concentration was found as adverse effects were reported more frequently in the patient group with supratherapeutic mean EFV concentrations compared to those with therapeutic EFV concentrations (p = 0.041). Correlations between EFV plasma concentrations and adverse effects, in both adults and children, have previously been published [46] and it is likely that supratherapeutic plasma concentrations cause additional drug-associated unwanted effects. No patient had a subtherapeutic mean EFV plasma concentration probably due to dosage correction in patients in whom occasional low EFV concentrations were observed.

In contrast to the statistically significant correlation between drug concentration and side effects, there was no significant correlation between drug concentration and viral load. This is contrary to the hypothesis that high levels leading to side effects could result in poor adherence and treatment failure. Low plasma concentrations have previously been related to treatment failure [35, 36]. There were too few patients with low EFV plasma levels in our study to allow analysis of such a relationship.

The dosage of EFV was adjusted during the course of treatment in 12 patients (29%) after evaluation of EFV plasma concentration and thus, the adjusted dose did not follow the treatment recommendations based on weight. Since the information about dose and weight was collected at the same occasion as the EFV plasma concentration and the outcome measure EFV plasma concentration/(dose/weight) was calculated, the risk of bias from dose correction was hopefully minimized in the study. Repeated measurements of EFV plasma concentrations and the adjustments of dosage thereafter is not a standard procedure but was beneficial to achieve long-term therapeutic EFV concentration, avoid side effects and possibly treatment failure due to high and low EFV plasma concentration in a multi-ethnic setting. The clinical data and documentation from this study provide important knowledge to current monitoring and dosing recommendations.

This is a retrospective study with the limitations related to such a design. The major concerns are the relevance and completeness of reported adverse effects and the correction of doses based on plasma concentration results. According to the latter, the outcome measure log_e_ concentration/(dose/weight) possibly compensate to the bias caused by this. The number of patients with ethnic origin other than African was low.

In conclusion, this retrospective study performed in routine care of patients confirmed major interindividual variability in EFV plasma concentration in pediatric HIV patients in a multi ethnic high-income setting. We found a clear genomic correlate of interindividual variation. The differences in EFV concentrations could be explained to a high extent (75%) by polymorphisms in drug metabolizing enzyme genes, mainly CYP2B6 and CYP2A6. Also, age at treatment initiation as well as time from treatment initiation were significant factors. Supratherapeutic mean EFV plasma concentration was related to adverse effects but EFV plasma concentration could not be related to treatment results. Individualized dosing guided by therapeutic drug monitoring and/or genotyping results in therapeutic EFV plasma concentrations and decreases the risk for interruptions in the ARV therapy. Thereby better treatment outcome can be expected in a clinical setting.

Knowledge of individual variants in key drug metabolizing enzymes, could improve the clinical safety and precision in achieving optimal plasma concentrations. This would make EFV treatment even more useful and sustainable in the lifelong treatment of HIV-infected children.

## Supporting information

S1 Dataset De-identified data set. (XLSX)
